# Temperature effects on incidence of surgery for acute type A aortic dissection in the Nordics

**DOI:** 10.1080/16549716.2022.2139340

**Published:** 2022-11-08

**Authors:** Daniel Oudin Åström, Henrik Bjursten, Anna Oudin, Shahab Nozohoor, Khalil Ahmad, Mariann Tang, Markus Bjurbom, Emma C Hansson, Anders Jeppsson, Christian Joost Holdflod Møller, Miko Jormalainen, Tatu Juvonen, Ari Mennander, Peter S Olsen, Christian Olsson, Anders Ahlsson, Emily Pan, Peter Raivio, Anders Wickbom, Johan Sjögren, Arnar Geirsson, Tomas Gudbjartsson, Igor Zindovic

**Affiliations:** a Division of Sustainable Health, Department of Public Health and Clinical Medicine, Umeå University, Umeå, Sweden; b Division of Occupational and Environmental Medicine, Department of Laboratory Medicine, Lund University, Lund, Sweden; c Department of Cardiothoracic Surgery, Skåne University Hospital, Lund University, Lund, Sweden; d Department of Cardiothoracic and Vascular Surgery, Aarhus University Hospital, Aarhus, Denmark; e Department of Thoracic and Cardiovascular Surgery, Karolinska University Hospital, Stockholm, Sweden; f Department of Molecular and Clinical Medicine, Institute of Medicine, Sahlgrenska Academy, University of Gothenburg, Gothenburg, Sweden; g Department of Cardiothoracic Surgery, Sahlgrenska University Hospital, Gothenburg, Sweden; h Department of Cardiothoracic Surgery, The Heart Centre, Rigshospitalet, Copenhagen University Hospital, Copenhagen, Denmark; i Heart and Lung Center, Helsinki University Hospital, Helsinki, Finland; j Research Unit of Surgery, Anesthesia, and Critical Care, University of Oulu, Oulu, Finland; k Heart Centre, Tampere University Hospital and University of Tampere, Tampere, Finland; l Department of Cardiothoracic Surgery, Centre for Cardiac, Vascular, Pulmonary and Infectious Diseases. Rigshospitalet, Copenhagen, Denmark; m Heart Center, Turku University Hospital, Turku, Finland; n Cardiovascular Medicine Division, Brigham and Women’s Hospital and Harvard Medical School, Boston, MA, USA; o Department of Cardiothoracic and Vascular Surgery, Orebro University Hospital, Orebro, Sweden; p Division of Cardiac Surgery, Yale University School of Medicine, New Haven, CT, USA; q Department of Cardiothoracic Surgery, Landspitali University Hospital and Faculty of Medicine, University of Iceland, Reykjavik, Iceland

**Keywords:** Heat, cold, temperature, acute type A aortic dissection, muli-centre study

## Abstract

We aimed to investigate a hypothesised association between daily mean temperature and the risk of surgery for acute type A aortic dissection (ATAAD). For the period of 1 January 2005 until 31 December 2019, we collected daily data on mean temperatures and date of 2995 operations for ATAAD at 10 Nordic cities included in the Nordic Consortium for Acute Type A Aortic Dissection (NORCAAD) collaboration. Using a two-stage time-series approach, we investigated the association between hot and cold temperatures relative to the optimal temperature and the rate of ATAAD repair in the selected cities. The relative risks (RRs) of cold temperatures (≤−5°C) and hot temperatures (≥21°C) compared to optimal temperature were 1.47 (95% CI: 0.72–2.99) and 1.43 (95% CI: 0.67–3.08), respectively. In line with previous studies, we observed increased risk at cold and hot temperatures. However, the observed associations were not statistically significant, thus only providing weak evidence of an association.

## Introduction

There is an extensive body of research concerning the impact of high and low ambient temperatures on mortality and morbidity [1–4]. The effect of heat on mortality usually corresponds with increases on the same day or one or two days after an increase in temperature, whereas cold-related mortality has been reported at longer time lags [1]. The temperature-mortality association is often described as a non-linear U-shaped curve, with a nadir temperature at which mortality is the lowest [2,3].

Cardiovascular heat- and cold-related mortality and morbidity have previously been observed in the Nordic region [5–13]. For instance, a history of hospitalisation for myocardial infarction increased the risk of dying in association with cold temperatures [12].

Given the known influence of temperature on other acute cardiovascular events, the association between temperature and the risk of acute type A aortic dissection (ATAAD) has previously been investigated. These studies were limited by small study samples and show conflicting results [14–19]. Recently, however, two large studies using advanced statistical methods reported increased risk of acute aortic dissection at lower temperatures [20,21].

Our aim in the present study was to investigate a hypothesised association between cold and hot temperatures and the risk of ATAAD repairs in a relatively homogenous Nordic cohort of patients and to assess whether the recent findings [20,21] are generalisable to a Nordic population.

## Materials and methods

### Data

For the time period of 1 January 2005 to 31 December 2019, we collected daily data on mean temperature and counts of ATAAD repairs for the 10 participating cities (Reykjavik, Iceland; Aarhus and Copenhagen, Denmark; Gothenburg, Lund, Stockholm, and Örebro, Sweden; and Helsinki, Tampere, and Turku, Finland). NORCAAD registry has been described in detail elsewhere [22].

### Statistical methods

The association between daily mean temperature and ATAAD was investigated through a well-established two-stage time-series approach [3]. In the first stage, as the independent variable is a count, we fit an overdispersed Poisson regression model for each city adjusting for national holidays. We used a distributed lag non-linear model considering lag times of up to 21 days, which has been shown to be sufficient to capture the immediate effects of heat as well as more delayed effects of cold temperatures [3]. For temperature and time lag, we fit a quadratic B-spline and a natural cubic spline, respectively. For temperature, two equally spaced internal knots were used, and for the lag structure, two equally spaced knots were placed on the log scale. We then pooled the city-specific overall cumulative estimated temperature-ATAAD association using a multivariate meta-analytical model. We chose not to include atmospheric pressure in our models as it is not a confounder in studies like this [23].

R version 4.0.3 [24] and packages DLNM and MVMETA were used for analyses and graphics [25,26].

## Results

A total of 2995 surgeries for ATAAD were performed at the participating centres during the study period. Table 1 shows the descriptive statistics on yearly counts of ATAAD operations and temperature distributions for the daily mean temperature for the included cities.

**Table 1.** Yearly median ATAADs and interquartile range (IQR) and temperature distributions for the daily mean temperature for the included cities.

City	Yearly number of ATAADs	Temperature °C (daily mean)		
	Median (IQR)	Min	5^th^/95^th^ percentile	Max
*DENMARK*				
Aarhus	15 (10–22)	−6.8	0.6/23.5	33.4
Copenhagen	44 (31–51)	−6.8	0.5/24.3	33.9
*FINLAND*				
Helsinki	22 (19–25)	−20.7	−4.8/23.6	33.2
Tampere	9 (5–11)	−23.8	−6.9/24.5	32.5
Turku	7 (5–10)	−20.9	−4.5/25.3	33.6
*ICELAND*				
Reykjavik	3 (2–4)	−10.2	−1.0/15.6	24.0
*SWEDEN*				
Göteborg	33 (30–35)	−10.1	−0.1/25.4	34.1
Lund	24 (22–28)	−8.9	0.2/26.2	34.4
Stockholm	29 (25–32)	−13.7	−2.2/25.2	31.9
Örebro	11 (9–14)	−16.9	−2.7/25.5	33.9

ATAAD: Acute Type A Aortic Dissection; IQR: Interquartile Range.

**Figure 1.**
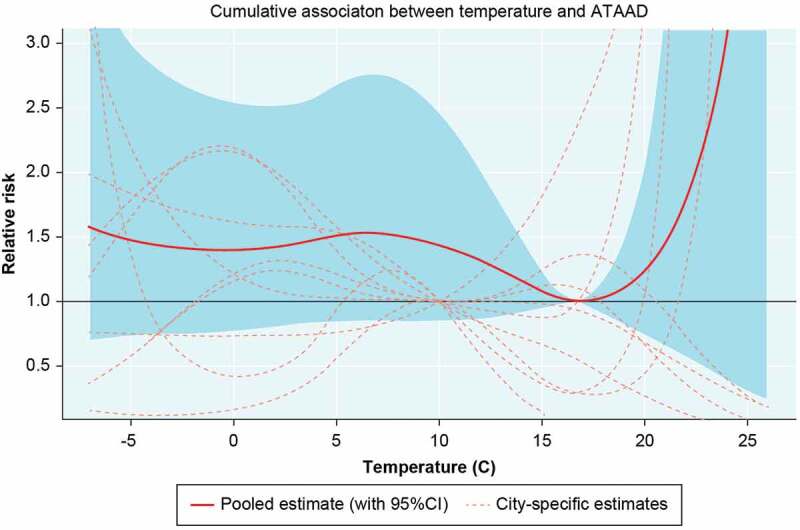
Pooled and city-specific estimates of the cumulative relative risks (RR) of ATAAD over 21 lags with 95% confidence intervals (grey area) in relation to daily mean temperature.

Figure 1 shows pooled and city-specific estimates of the cumulative relative risks (RR) over 21 lags for the temperature-ATAAD repair association. The optimal temperature across the countries is a daily mean temperature of approximately 17°C, meaning that the risk of ATAAD repairs was lowest at that temperature. Increasing risks were observed at both ends of the temperature distribution, however, not to a statistically significant extent. The RRs comparing the optimal temperature (17°C) to cold temperatures (−5°C) and hot temperatures (21°C) were 1.47 (95% CI: 0.72–2.99) and 1.43 (95% CI: 0.67–3.08), respectively.

## Discussion

In this study, our pooled results indicate a tendency towards increased risk of ATAAD at cold and hot temperatures in the Nordic setting but not to a statistically significant extent. Albeit non-significant, the risk increase at cold temperatures is in line with recent findings (20, 21).

It is well-established that hot and cold temperatures induce a wide variety of physiological responses, leading to increased cardiovascular stress [27–29]. As previously mentioned, most studies on the relationship between temperature and ATAAD risk have been characterised by small study samples. Repanos et al. and Majd et al. found no effect of temperature on risk of ATAAD in 26 and 348 patients [17,19]. Benouaich et al. reported higher rates of ATAAD in winter than summer in 206 patients and a correlation between temperature drop three days preceding symptoms and ATAAD [18]. Verberkmoes et al. showed a correlation between daily minimum temperature and incidence of ATAAD in 212 patients, suggesting a higher incidence on colder days [15]. In addition, in a larger study of 3000 patients with aortic dissection (not specified as type A or B), Law et al. reported a lower incidence of aortic dissection at higher temperatures [16].

Yu et al., using a time-series approach considering lag times of up to 25 days, recently reported a relative risk of 1.7 (95% CI: 1.1–1.7) and 4.0 (1.7–9.4) for AAD and ATAAD, respectively, at extremely cold temperatures (0.5°C) compared to the reference temperature of 25°C [20]. In addition, Chen et al. reported odds ratios of 2.8 (95% CI: 1.7–4.8) at extremely cold temperatures (−10°C) and of 2.4 (95% CI: 1.6–3.5) at cold temperatures (1°C) [21].

The shape of the exposure–response function was in line with previously reported results for total mortality [3,30] and for AAD [20,21]. Furthermore, the relatively high optimal temperature, in our case 17°C, is in line with that in Yu et al. and Chen et al [20,21]. In contrast to the two mentioned studies, however, we observed a tendency of heat to increase the risk of ATAAD, which warrants further investigations in other settings.

Hot and cold temperatures induce a wide variety of physiological responses, leading to increased cardiovascular stress [27–29]. When exposed to high ambient temperatures, the human body attempts to regulate the interior temperature by increasing heart rate and cardiac output to redistribute blood to the skin where heat is dissipated by sweating, leading to increased pressure on heart and lungs [27]. The body’s response to exposure to low temperatures is to reduce heat loss from the body’s core by decreasing peripheral blood circulation, concentrating the blood. This promotes viscosity and hypercoagulability and increases blood pressure [29].

Heat exposure is reported to be associated with the increased heart rate and decreased blood pressure, whereas cold exposure is associated with increased blood pressure [31] as there is a negative correlation between blood pressure and daily mean temperature [32–34]. Recently, a review and meta-analyses of 47 studies reported strong evidence for changes in blood pressure between seasons, with the largest effects found in the elderly and patients treated for hypertension [35]. Thus, the variability in blood pressure caused by deviation from the optimal temperature of 17°C may increase the risk of ATAAD.

### Strengths and limitations

One of the main strengths of the current study is the long and well-defined time series of daily temperature and aortic dissection data from the 10 cities. The number of missing meteorological observations also was very low. An additional strength is the statistical methodology used, allowing pooling of the different time series to generate a pooled estimate of the association between temperature and ATAAD for the participating Nordic countries. A limitation of the present study is that a single monitoring station was used in the city where the patient was operated on, not where the dissection occurred, so results may not reflect the exact temperature. In addition, for some centres, the catchment areas are very large, and naturally, the temperature can differ, causing exposure misclassification. The approach used, however, is a standard method for reporting on temperature and various health outcomes, so the misclassification is most likely non-differential [36]. In addition, individuals surviving long enough to have surgery may have a different disease pathology from those dying in an out-of-hospital setting. Another limitation is that, despite NORCAAD being among the largest available cohorts on ATAAD, the daily number of dissections is low, resulting in a low statistical power to detect differences across the temperature distribution. Finally, the study is isolated to centres from Northern Europe, and although the homogeneity of the study population strengthens this study, the physiological effects of variation in temperatures may not be generalisable to other climate zones.

## Conclusions

The risk of surgery for acute type A aortic dissection tended to increase at cold temperatures in a Nordic setting, which is in line with previous findings. Our results, however, were not statistically significant, suggesting cautious interpretation of the temperature-ATAAD association in the Nordic region, and may warrant further research.

## Paper context

There is suggestive evidence of the impacts of high and low temperatures on mortality and morbidity; however less is known about the impact on the risk of surgery for acute type A aortic dissection. Applying time series regression for ten cities in the Nordics and pooling the results with a meta-analytic approach, we observed weak evidence of higher risk of surgery at high and low temperatures, however, not to a statistically significant extent.

## References

[1] Anderson BG, Bell ML. Weather-related mortality: how heat, cold, and heat waves affect mortality in the United States. Epidemiology. 2009;20:205. 19194300 10.1097/EDE.0b013e318190ee08 PMC3366558

[2] Guo Y, Gasparrini A, Armstrong B, Li S, Tawatsupa B, Tobias A, et al. Global variation in the effects of ambient temperature on mortality: a systematic evaluation. Epidemiology. 2014;25:781–5. 25166878 10.1097/EDE.0000000000000165 PMC4180721

[3] Gasparrini A, Guo Y, Hashizume M, Lavigne E, Zanobetti A, Schwartz J, et al. Mortality risk attributable to high and low ambient temperature: a multicountry observational study. Lancet. 2015;386:369–375. 26003380 10.1016/S0140-6736(14)62114-0 PMC4521077

[4] Åström DO, Ebi KL, Vicedo-Cabrera AM, Gasparrini A. Investigating changes in mortality attributable to heat and cold in Stockholm, Sweden. Int J Biometeorol. 2018;62:1777–1780. 29748912 10.1007/s00484-018-1556-9 PMC6132879

[5] Oudin Åström D, Åström C, Forsberg B, Vicedo-Cabrera AM, Gasparrini A, Oudin A, et al. Heat wave–related mortality in Sweden: a case-crossover study investigating effect modification by neighbourhood deprivation. Scand J Public Health. 2020;48:428–435. 30253698 10.1177/1403494818801615 PMC6713612

[6] Wichmann J, Andersen ZJ, Ketzel M, Ellermann T, Loft S. Apparent temperature and cause-specific mortality in Copenhagen, Denmark: a case-crossover analysis. Int J Environ Res Public Health. 2011;8:3712–3727. 22016711 10.3390/ijerph8093712 PMC3194112

[7] Wichmann J, Ketzel M, Ellermann T, Loft S. Apparent temperature and acute myocardial infarction hospital admissions in Copenhagen, Denmark: a case-crossover study. Environ Health. 2012;11:19. 22463704 10.1186/1476-069X-11-19 PMC3353865

[8] Sohail H, Tiittanen P, Kollanus V, Lanki T. Heat, heatwaves and cardiorespiratory hospital admissions in Helsinki, Finland. Eur J Public Health. 2020;30:ckaa166.40. 10.3390/ijerph17217892 PMC7663418 33126485

[9] Group TE. Cold exposure and winter mortality from ischaemic heart disease, cerebrovascular disease, respiratory disease, and all causes in warm and cold regions of Europe. Lancet. 1997;349:1341–1346. 9149695

[10] Pfeifer K, Oudin Åström D, Martinsone Ž, Kaļužnaja D, Oudin A. Evaluating mortality response associated with two different Nordic heat warning systems in Riga, Latvia. Int J Environ Res Public Health. 2020;17:7719. 10.3390/ijerph17217719 PMC7672594 33105717

[11] Åström DO, Veber T, Martinsone Ž, Kaļužnaja D, Indermitte E, Oudin A, et al. Mortality related to cold temperatures in two capitals of the Baltics: Tallinn and Riga. Medicina (B Aires). 2019;55:429. 10.3390/medicina55080429 PMC6723676 31382432

[12] Rocklöv J, Forsberg B, Ebi K, Bellander T. Susceptibility to mortality related to temperature and heat and cold wave duration in the population of Stockholm County, Sweden. Glob Health Action. 2014;7:22737. 24647126 10.3402/gha.v7.22737 PMC3955771

[13] Orru H, Åström DO. Increases in external cause mortality due to high and low temperatures: evidence from northeastern Europe. Int J Biometeorol. 2017;61:963–966. 27858164 10.1007/s00484-016-1270-4 PMC5411405

[14] Shahraiyni HT, Sodoudi S, Cubasch U. Weather conditions and their effect on the increase of the risk of type a acute aortic dissection onset in Berlin. Int J Biometeorol. 2016;60:1303–1305. 26546312 10.1007/s00484-015-1099-2

[15] Verberkmoes N, Hamad MS, Ter Woorst J, Tan M, Peels C, van Straten A. Impact of temperature and atmospheric pressure on the incidence of major acute cardiovascular events. Netherlands Heart J. 2012;20:193–196. 10.1007/s12471-012-0258-x PMC3346877 22328355

[16] Law Y, Chan Y, Cheng S. Influence of meteorological factors on acute aortic events in a subtropical territory. Asian J Surg. 2017;40:329–337. 26857853 10.1016/j.asjsur.2015.11.002

[17] Majd P, Madershahian N, Sabashnikov A, Weber C, Ahmad W, Weymann A, et al. Impact of meteorological conditions on the incidence of acute aortic dissection. Ther Adv Cardiovasc Dis. 2018;12:321–326. 30244647 10.1177/1753944718801559 PMC6266247

[18] Benouaich V, Soler P, Gourraud PA, Lopez S, Rousseau H, Marcheix B. Impact of meteorological conditions on the occurrence of acute type a aortic dissections. Interact Cardiovasc Thorac Surg. 2010;10:403–406. 20008897 10.1510/icvts.2009.219873

[19] Repanos C, Chadha NK. Is there a relationship between weather conditions and aortic dissection? BMC Surg. 2005;5:21. 16225700 10.1186/1471-2482-5-21 PMC1266384

[20] Yu X, Xia L, Xiao J, Zheng J, Xu N, Feng X, et al. Association of daily mean temperature and temperature variability with onset risks of acute aortic dissection. J Am Heart Assoc. 2021;10:e020190. 34169738 10.1161/JAHA.120.020190 PMC8403292

[21] Chen J, Gao Y, Jiang Y, Li H, Lv M, Duan W, et al. Low ambient temperature and temperature drop between neighbouring days and acute aortic dissection: a case-crossover study. Eur Heart J. 2022;43:228–235. 34849712 10.1093/eurheartj/ehab803

[22] Geirsson A, Ahlsson A, Franco-Cereceda A, Fuglsang S, Gunn J, Hansson EC, et al. The Nordic Consortium for acute type a aortic dissection (NORCAAD): objectives and design. Scan Cardiovasc J. 2016;50:334–340. 10.1080/14017431.2016.1235284 27615395

[23] Buckley JP, Samet JM, Richardson DB. Commentary: does air pollution confound studies of temperature? Epidemiology. 2014;25:242–245. 24487206 10.1097/EDE.0000000000000051

[24] R Core Team (2022). R: A language and environment for statistical computing. R Foundation for Statistical Computing, Vienna, Austria. URL https://www.R-project.org/.

[25] Gasparrini A. Distributed lag linear and non-linear models in R: the package dlnm. J Stat Softw. 2011;43:1. PMC3191524 22003319

[26] Gasparrini A, Armstrong B, Kenward MG, Multivariate meta-analysis for non-linear and other multi-parameter associations. Stat Med. 2012;31:3821–3839. 22807043 10.1002/sim.5471 PMC3546395

[27] Worfolk JB. Heat waves: their impact on the health of elders. Geriatric Nurs. 2000;21:70–77. 10.1067/mgn.2000.107131 10769330

[28] Ebi KL, Mills D. Winter mortality in a warming climate: a reassessment. Wiley Interdis Rev Clim Change. 2013;4:203–212.

[29] Valtonen RI, Kiviniemi A, Hintsala HE, Ryti NR, Kenttä T, Huikuri HV, et al. Cardiovascular responses to cold and submaximal exercise in patients with coronary artery disease. Am J Physiol Regul Integr Comp Physiol. 2018;315:R768–76. 29975565 10.1152/ajpregu.00069.2018

[30] Åström DO, Tornevi A, Ebi KL, Rocklöv J, Forsberg B. Evolution of minimum mortality temperature in Stockholm, Sweden, 1901–2009. Environ Health Perspect. 2016;124:740. 26566270 10.1289/ehp.1509692 PMC4892916

[31] Hu S, Maeda T. Productivity and physiological responses during exposure to varying air temperatures and clothing conditions. Indoor Air. 2020;30:251–263. 31755604 10.1111/ina.12628

[32] Kuneš J, Tremblay J, Bellavance F, Hamet P. Influence of environmental temperature on the blood pressure of hypertensive patients in Montreal. Am J Hypertens. 1991;4:422–426. 2069775 10.1093/ajh/4.5.422

[33] Barnett AG, Sans S, Salomaa V, Kuulasmaa K, Dobson AJ, Project WM. The effect of temperature on systolic blood pressure. Blood Press Monit. 2007;12:195–203. 17496471 10.1097/MBP.0b013e3280b083f4

[34] Alpérovitch A, Lacombe J-M, Hanon O, Dartigues J-F, Ritchie K, Ducimetière P, et al. Relationship between blood pressure and outdoor temperature in a large sample of elderly individuals: the Three-City study. Arch Internal Med. 2009;169:75–80. 19139327 10.1001/archinternmed.2008.512

[35] Kollias A, Kyriakoulis KG, Stambolliu E, Ntineri A, Anagnostopoulos I, Stergiou GS. Seasonal blood pressure variation assessed by different measurement methods: systematic review and meta-analysis. J Hypertens. 2020;38:791–798. 32102047 10.1097/HJH.0000000000002355

[36] Basu R. High ambient temperature and mortality: a review of epidemiologic studies from 2001 to 2008. Environ Health. 2009;8:1–13. 19758453 10.1186/1476-069X-8-40 PMC2759912

